# Switching to PegIFNα-2b leads to HBsAg loss in patients with low HBsAg levels and HBV DNA suppressed by NAs

**DOI:** 10.1038/s41598-017-13747-9

**Published:** 2017-10-17

**Authors:** Jing Huang, Ka Zhang, Wenli Chen, Jinyao Liao, Xiaodan Luo, Ren Chen

**Affiliations:** 1 Department of Infectious Diseases, Guangdong General Hospital (Guangdong Academy of Medical Sciences), Guangzhou, China; 2 Department of Infectious Diseases, The 3rd Affiliated Hospital, Sun Yat-Sen University, Guangzhou, China

## Abstract

Patients with low hepatitis B surface antigen (HBsAg) levels and hepatitis B virus (HBV) DNA suppression by nucleos(t)ide analogues (NAs) achieve high rate of HBsAg loss through switching to PegIFNα in pre-registration study. The aim of this study was to achieve higher rate of HBsAg loss through extended PegIFN treatment. 98 patients with HBsAg < 2,000 IU/ml and HBV DNA < 20 IU/ml were randomized to receive PegIFNα-2b or continuing NA therapy for 60 weeks. At the end of treatment (EOT) and end of follow-up (EOF), only patients who switched to PegIFNα-2b achieved HBsAg loss (32.6%) and HBsAg seroconversion (27.9% and 25.6%). Patients who switched to PegIFNα-2b also achieved higher HBeAg seroconversion rates (65.1%) and HBeAg loss (81.4% and 90.7%) than those who continued NAs treatment. On-treatment HBsAg declines predicted the responses at EOT, and HBsAg declines at post-baseline times predicted the responses at EOF. The rates of responses were not increased through extended PegIFNα treatment. For patients with low HBsAg and HBV suppression with NAs, switching to PegIFNα-2b significantly increased the rates of HBsAg loss and HBsAg seroconversion. HBsAg decline can predict the response of switching to PegIFNα-2b following from NAs.

## Introduction

Although safe and effective prophylactic vaccines have been available for the past 30 years, hepatitis B virus (HBV) infection remains an important public health problem and is the leading cause of chronic hepatitis B (CHB), cirrhosis and hepatocellular carcinoma (HCC) worldwide^1^. The current antiviral treatments for CHB include nucleos(t)ide analogues (NAs) and peginterferon (PegIFN) alfa. NAs can effectively suppress the HBV viral loads and reduce HBV-related morbidity and mortality^2^. However, the virological and clinical relapse rates are high after cessation of treatment; so long-term treatment is required^3^. PegIFN alfa can induce viral suppression in approximately 25% of patients but is associated with additional side effects^4–6^. Currently, hepatitis B surface antigen (HBsAg) loss within a finite duration of therapy is considered a “functional cure” but is difficult to obtain through current antiviral approaches. Thus, a therapy leading to HBsAg loss with the aim to end treatment is highly desirable.

Previous studies have reported that the serum HBsAg level is related to intrahepatic covalently closed circular DNA (cccDNA)^7^, and serum HBsAg lowered to an undetectable level may indicate that intrahepatic cccDNA has been eradicated^8,9^. In addition, a reduction in the serum HBsAg level predicts serological and clinical outcomes and treatment responses^10–13^. Hence, HBsAg loss is the ideal endpoint for antiviral therapy. Unfortunately, the incidence of HBsAg loss is low with the currently available CHB treatments, at 10% to 12% for those patients receiving long-term follow-up treatment with both peg-IFN and NAs^14–16^. As PegIFN alfa and NAs have different and complementary routes of action, the therapeutic approach of combining both drugs might be a good option for the treatment of CHB. Adding PegIFN to entecavir (ETV) significantly increased the reductions in HBsAg, hepatitis B e antigen (HBeAg), and HBV DNA and led to more sustained responses after ETV discontinuation, thereby preventing relapse after stopping NAs^17^. In one prospective study^18^, HBeAg-positive CHB patients who had received ETV for 9–36 months were randomized1:1 to switch to PegIFN alfa-2a or to continue with ETV for 48 weeks. The results showed that patients achieved virological suppression with ETV and that switching to a finite course of PegIFNalfa-2a significantly increased the rates of HBeAg seroconversion and HBsAg loss. Interestingly, the combination of HBeAg loss and HBsAg < 1,500 IU/ml at the time of switching was associated with high rates of HBeAg seroconversion (33.3%) and HBsAg loss (22.2%) following PegIFN alfa-2a therapy. In contrast, HBsAg levels > 1,500 IU/ml were associated with the lowest rate of response. Unfortunately, the number of patients with HBeAg loss and HBsAg < 1,500 IU/ml were limited, and the treatment duration of PegIFN treatment was only 48 weeks. Several studies have demonstrated the potential benefit of extending the interferon treatment duration^19^. However, extending the PegIFN treatment duration did not show superiority over 48 weeks of PegIFN monotherapy in one randomized controlled study; patients who achieved HBsAg < 1,500 IU/ml after 24 weeks of PegIFN alfa-2a showed satisfactory outcomes after the withdrawal of PegIFN alfa-2a treatment^20^. Nevertheless, all of above studies testing alternate optimization strategies were conducted on overall treatment-naïve patients and did not take into account NA-experienced patients. In some studies^18,21^, patients with HBsAg < 1,500 IU/ml showed high rates of HBeAg seroconversion and HBsAg loss, although these studies were not performed in separate cohorts of patients with HBsAg < 2,000 IU/ml and HBV DNA < 20 IU/ml. In addition, the extension of PegIFN treatment was not performed, although longer treatment durations may lead to higher rates of HBsAg loss. Finally, these previous studies did not feature long-term follow-up after the cessation of the treatment or comparison with NA treatment alone.

The aim of the current study was to prospectively evaluate whether the extension of switching to PegIFN from a stable NA regimen leads to a high loss of HBsAg in CHB patients with HBsAg < 2,000 IU/ml and HBV DNA < 20 IU/ml by nucleos(t)ide analogues.

## Results

### Patient characteristics

A total of 146 CHB patients with at least 2 years of prior NA exposure, HBsAg < 2,000 IU/ml and serum HBV DNA levels < 20 IU/ml were enrolled. Patients were randomized into 2 treatment groups and matched as pairs at a 1:1 ratio in terms of gender, age, duration of previous treatment with NAs and baseline HBsAg and alanineaminotransferase (ALT) levels. Ultimately, the matched-pair set consisted of 47 patients treated with PegIFN alfa-2b therapy (group A) and 47 patients treated with NA monotherapy (group B). Of these patients, 4 patients discontinued treatment due to loss to follow-up (2 in group A, 2 in group B), patient request (1 in group A), and side effects (1 in group A). In total, 88 patients completed at least 60 weeks of treatment. Table 1 shows the age, sex, and baseline clinical and viral characteristics of these patients. These variables were generally similar among the 2 groups, and no significant differences were observed in the baseline characteristics between the groups.

**Table 1:** Patient demographics and baseline characteristics.

Baseline characteristic	PegIFNalfa-2b (n = 43)	NAs (n = 45)	P value
Males, n (%)	30 (69.8)	35 (77.8)	0.393
age, yr, Mean (SD)	32.26 ± 6.81	32.20 ± 6.96	0.969
Duration of NAs treatment, yr, Mean (SD)	3.44 ± 1.25	3.64 ± 0.87	0.389
ALT, U/L, Mean (SD)	27.12 ± 13.45	23.00 ± 7.46	0.078
HBsAg, Mean (SD)IU/ml	1,030.6 (285.2,1,507.0)	991.2 (593.2,1,459.0)	0.796
AST, U/L, Mean (SD)	23.0 ± 12.5	21 ± 10	0.186
HBeAg loss n (%)	12 (27.9%)	10 (22.2%)	0.538
HBeAg seroconversion n (%)	8 (18.6%)	7 (15.56%)	0.923

HBeAg, hepatitis B e antigen; HBsAg, hepatitis B surface antigen; NAs, nucleos(t)ide analogues; ALT, alanineaminotransferase.

### Response to treatment at weeks 24, 48, and 60

At weeks 24, 48 and 60, patients in group A achieved higher rates of HBsAg loss, HBeAg loss and HBeAg seroconversion than those in the NA monotherapy (group B) (P < 0.05; Table 2). Meanwhile, conversely, the levels of HBsAg in group A at weeks 24, 48 and 60 were lower than those of group B (P = 0.000; Table 2). In total, after 60 weeks of treatment, significantly more patients in the PegIFN alfa-2b therapy group (14/43, 32.6%) achieved the primary endpoint of HBsAg loss than those in the NA monotherapy group (0, 0%; P = 0.000; Table 2). HBsAg seroconversion at week 60 occurred in 12 patients (12/43, 27.9%) treated with PegIFN alfa-2b therapy, but was not achieved in patients treated with NA monotherapy (P = 0.000; Table 2). Ten of 43 (24.39%) patients in the PegIFN alfa-2b therapy group achieved HBsAg seroconversion after 48 weeks of treatment, and the difference between the two groups was statistically significant (P = 0.000; Table 2).However, similar rates of HBsAg seroconversion were achieved in the two groups at week 24 (P = 0.236; Table 2). Surprisingly, the levels of ALT in group A at weeks 24, 48 and 60 were higher than those of group B (P = 0.000; Table 2).

**Table 2:** Comparison of on-treatment response rates between the switch to PegIFN alfa-2b therapy and NA monotherapy.

Outcome	Week 24			Week 48			Week 60		
	PegIFN alfa-2b	NAs	P value	PegIFN alfa-2b	NAs	P value	PegIFN alfa-2b	NAs	P value
**ALT (U/ml)**	62.53 (41,74)	24.22 (16,30)	0.000	59.12 (40.75,73)	24.34 (19,29.75)	0.000	44.53 (41,74)	23.53 (35.5,51)	0.000
**HBsAg (log IU/ml)**	1.46 ± 1.66	2.89 ± 0.39	0.000	0.97 ± 1.70	2.85 ± 0.41	0.000	0.1 ± 1.24	2.8 ± 0.46	0.000
**HBsAg loss n (%)**	8 (18.6%)	0 (0%)	0.002	11 (26.83%)	0 (0%)	0.001	14 (32.6%)	0 (0%)	0.000
**HBsAg seroconversion n (%)**	2 (4.65%)	0 (0%)	0.236	10 (24.39%)	0 (0%)	0.000	12 (27.9%)	0 (0%)	0.000
**HBeAg loss n(%)**	23 (53.5%)	10 (22.22%)	0.002	31 (72.7%)	11 (24.4%)	0.000	35 (81.4%)	12 (26.7%)	0.000
**HBeAg seroconversion n (%**)	21 (48.8%)	9 (20.0%)	0.004	23 (53.5%)	8 (17.8%)	0.000	28 (65.1%)	6 (13.3%)	0.000

HBeAg, hepatitis B e antigen; HBsAg, hepatitis B surface antigen; NAs, nucleos(t)ide analogues; ALT, alanineaminotransferase

### Response to treatment at week 84 and 108 (follow-up for 24 weeks and 48 weeks)

At weeks 84 (24 weeks of follow-up)and 108 (48 weeks of follow-up), patients treated with PegIFN alfa-2b therapy (group A)still showed higher rates of treatment responses (HBsAg seroconversion, HBsAg loss, HBeAg seroconversion, and HBeAg loss) than those in group B (P = 0.000; Table 3). The levels of HBsAg in group A at weeks 84 and 106 were lower than those of group B (P = 0.000; Table 3). By the end of follow-up (EOF) (108 weeks), significantly more patients in the PegIFN alfa-2b therapy group (14/43, 32.6%)achieved the primary endpoint of HBsAg loss than in the NA monotherapy group (0, 0%; P = 0.000; Table 3). Eleven patients (25.6%) treated with PegIFN alfa-2b therapy achieved HBsAg seroconversion (secondary endpoint) at EOF, while this was not achieved in patients treated with NA monotherapy (P = 0.000; Table 3).

**Table 3:** Comparison of off-treatment response rates between the switch to PegIFN alfa-2b therapy and NA monotherapy.

Outcome	Week 84			Week 108		
	PegIFN alfa-2b	NAs	P value	PegIFN alfa-2b	NAs	P value
**ALT (U/ml)**	30.36 (22.75,33.0)	22.87 (16.50,29.0)	0.004	33.40 (18.75,32.0)	29.43 (16.25,29.0)	0.139
**HBsAg (log IU/ml)**	10.90 (0.05,670.2)	694.3 (331.5,1140.5)	0.000	9.77 (0.05,723.35)	685.3 (328.2,1037.3)	0.000
**HBsAg lossn (%)**	13 (30.2%)	0 (0%)	0.000	14 (32.6%)	0 (0%)	0.000
**HBsAg seroconversion n (%)**	13 (30.2%)	0 (0%)	0.000	11 (25.6%)	0 (0%)	0.000
**HBeAg loss n (%)**	34 (79.1%)	17 (37.8%)	0.000	39 (90.7%)	17 (37.8%)	0.000
**HBeAg seroconversion n (%)**	25 (58.1%)	7 (15.6%)	0.000	28 (65.1%)	10 (22.2%)	0.000

HBeAg, hepatitis B e antigen; HBsAg, hepatitis B surface antigen; NAs, nucleos(t)ide analogues; ALT, alanineaminotransferase.

### Response rates changes according to observation week

**Figure 1:**
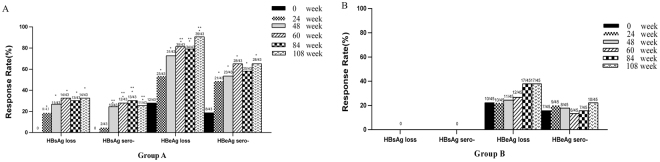
Comparison of response rates among different observation weeks. HBeAg, hepatitis B e antigen; HBsAg, hepatitis B surface antigen.

Response rates varied based on the week of observation in the PegIFN alfa-2b therapy group. The rates of HBsAg seroconversion, HBsAg loss, HBeAg seroconversion, and HBeAg loss at all post-baseline time points were significantly greater than those at baseline in the PegIFN alfa-2b therapy group (Fig. 1A). The rates of HBsAg seroconversion decreased at weeks 48, 60, 84 and 108 in the PegIFNalfa-2b therapy group and remained stable (Fig. 1A). The rate of HBeAg loss at weeks 60, 84 and 108 in the PegIFN alfa-2b therapy group were significantly greater than those at week 24 and remained stable at that level (Fig. 1A). However, these differences were not found in the NA therapy group (Fig. 1A).

### Baseline factors associated with treatment responses in group A

At EOT (week 60) and EOF (week 108), univariate analysis showed that baseline factors showed no significant differences among patients with responses or no responses in group A, including HBsAg seroconversion, HBsAg loss, HBeAg seroconversion, and HBeAg loss (P > 0.05). However, patients with HBsAg loss at week 24 were younger than patients with no HBsAg loss (27. 25 years versus 33. 40 years, P = 0.019).

### HBsAg declines according to the treatment regimen

**Figure 2:**
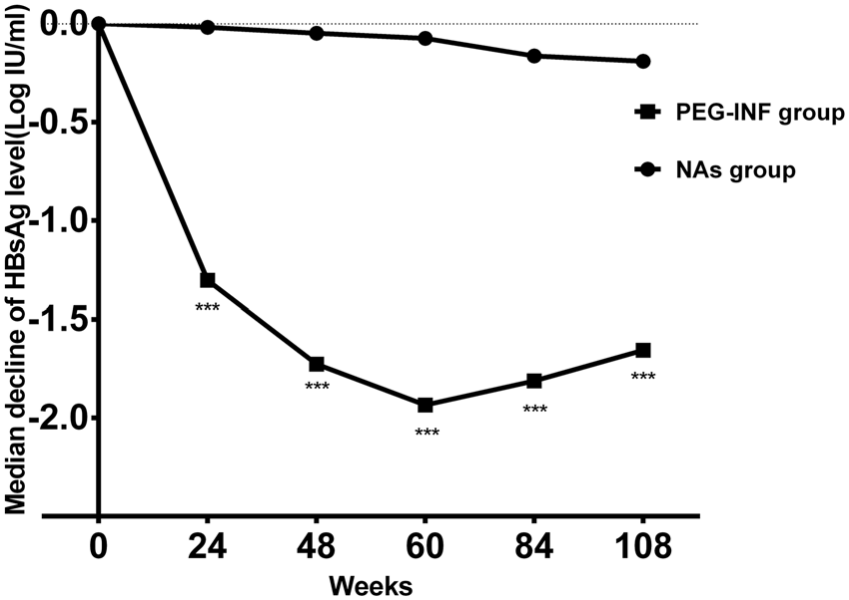
Comparison of the median declines in HBeAg levels between the switch to PegIFN alfa-2b therapy and NA monotherapy groups at different observation weeks. NAs, nucleos(t)ide analogues. ***P = 0.000.

The decline in HBsAg varied significantly according to the treatment regimen. The median serum HBsAg decline in the PegIFN alfa-2b therapy group was significantly greater than that in the NA group at all post-baseline time points (P = 0.000, Fig. 2). After 60 weeks of PegIFN alfa-2b therapy, viral rebound occurred in group A. However, patients in the PegIFN alfa-2b therapy (group A) continued to have higher HBsAg decline levels than patients in group B (P = 0.000; Fig. 2).

### Correlation between on-treatment HBsAg declines and treatment responses in patients treated with PEG-IFN alfa-2b at EOT

**Figure 3:**
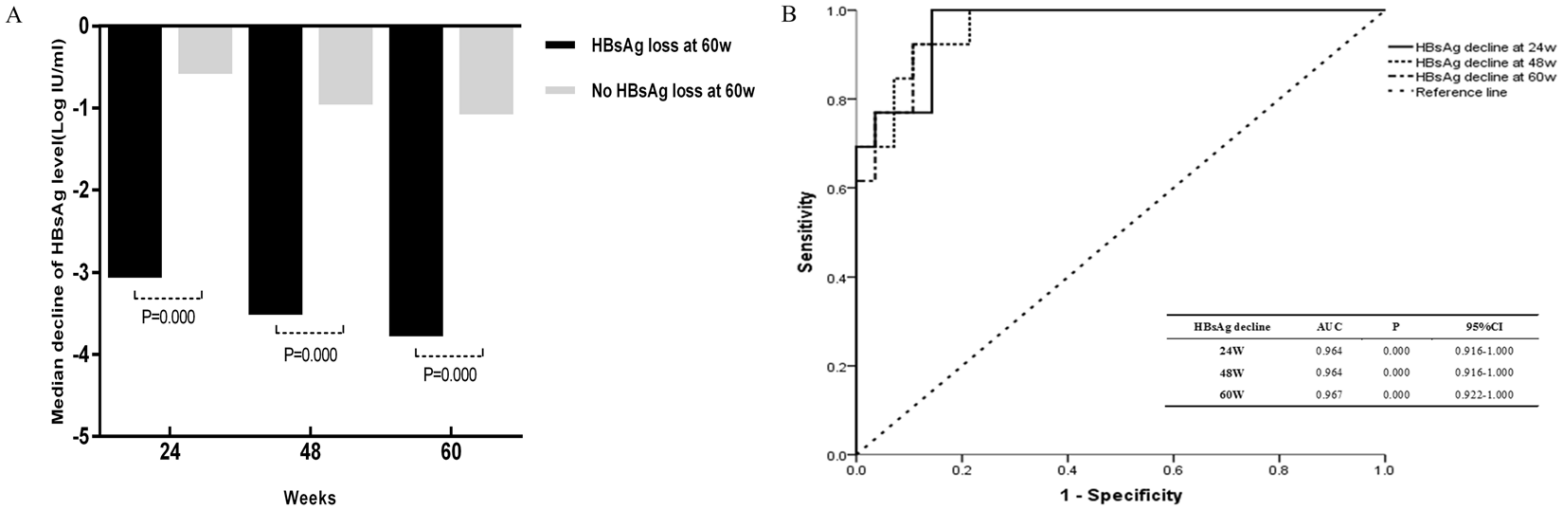
Comparison of on-treatment HBeAg declines between patients with HBsAg loss and no HBsAg loss (**A**) at EOT. ROC curves of on-treatment HBeAg declines for the prediction of HBsAg loss (**B**) at EOT. HBsAg, hepatitis B surface antigen; EOT: end of treatment; ROC, receiver-operating characteristic.

Among patients treated with PegIFN alfa-2b therapy, the median decline in the HBsAg level from baseline in patients who achieved HBsAg loss at week 60 was greater than that in patients who did not achieve HBsAg loss at weeks 24, 48 and 60 (Fig. 3A). ROC analysis confirmed that HBsAg declines at week 24, week 48 and week 60 were significantly associated with responses to PegIFN alfa-2b therapy (Fig. 3B). The AUCs were 0.964 (95% CI, 0.916–1.000) at week 24, 0.964 (95% CI, 0.916–1.000) at week 48, and 0.967 (95% CI, 0.922–1.000) at week 60 for prediction of HBsAg loss.

### Correlation between HBsAg declines and treatment responses in patients treated with peg-IFN alfa-2b at EOF

**Figure 4:**
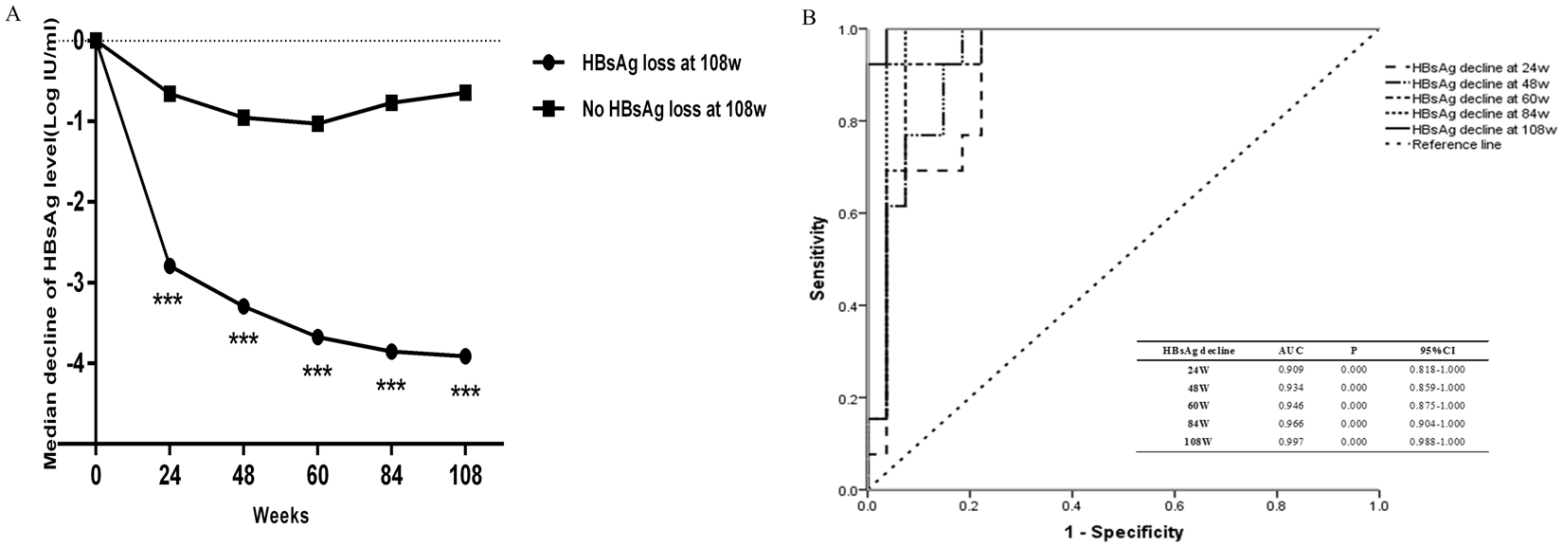
Comparison of HBeAg declines between patients with HBsAg loss and no HBsAg loss (**A**) at EOF.ROC curves of HBeAg declines for the prediction of HBsAg loss (**B**) at EOF. HBsAg, hepatitis B surface antigen; EOF: end of follow-up; ROC, receiver-operating characteristic. ***P = 0.000.

At EOF, a partial rebound of quantitative HBsAg from EOT to EOF was observed in group A after the discontinuance of peg-IFN alfa-2b (Fig. 1).The rates of HBsAg loss and seroconversion were still higher at EOF (32.6% and 25.6%). When we combined the median decline of HBsAg levels from baseline in patients who achieved HBsAg loss or no HBsAg loss, for patients with HBsAg loss at EOF, the HBsAg decline was significantly higher than that of patients with no HBsAg loss at all post baseline time points (P = 0.000, Fig. 4A). ROC analysis also confirmed that HBsAg declines at all post baseline time points was significantly associated with responses to peg-IFN alfa-2b therapy (Fig. 4B). The AUCs were 0.909 (95% CI, 0.818–1.000) at week 24, 0.934 (95% CI, 0.859–1.000) at week 48, 0.946 (95% CI, 0.875–1.000) at week 60, 0.966 (95% CI, 0.904–1.000) at week 84, and 0.997 (95% CI, 0.988–1.000) at week 108 for prediction of HBsAg loss.

Changes of ALT levels during treatment and off treatment period.

ALT elevations were observed early after switching to PegIFN alfa-2b in the patients with sustained HBV DNA suppression by NAs. At weeks 24, 48 and 60,the levels of ALT for patients switched to PegIFN alfa-2b was higher than that of patients continued to NAs (P < 0.05; Table 2). At EOF, the ALT levels of patients in group A was only higher than that of patients in group B at week 84 (P < 0.05; Table 2), and there was no difference between two groups at week 108 (P > 0.05; Table 2).

### Safety and tolerability

Switching from NAs to PegIFN therapy was generally well tolerated. No side effects related to this treatment switch were reported. The most common side effects in the switch arm were those known to occur with PegIFN therapy; the most frequent treatment-related side effects consisted of abnormal laboratory results, such as decreased white blood cell and neutrophil counts. No unexpected side effects were reported. One patient in the PegIFN alfa-2b therapy group requested treatment for persistent fever before week 24, and he did not complete the treatment. One patient assigned to the PegIFN alfa-2b therapy group experienced thyroid dysfunction, and she also did not complete the treatment.

## Discussion

Although curing chronic HBV infection is a challenging goal, recent advances in therapeutic approaches are making a cure possible. Previous studies have reported that the serum HBsAg level has some relationship with intrahepatic cccDNA, and serum HBsAg at an undetectable level may indicate that intrahepatic cccDNA has been eradicated^7,22^. HBsAg loss with or without seroconversion to anti-HBs antibodies is considered a functional cure and an endpoint where antiviral treatment can be stopped for CHB patients^23^. Recently, more studies^18,21^ have reported that switching treatment from NAs to PegIFN may induce higher rates of HBsAg loss. CHB patients who achieved virological suppression by ETV and switched to PegIFNalfa-2a for 48 weeks showed significantly increased rates of HBsAg loss (8.5%). Additionally, patients with HBsAg levels < 1,500 IU/ml by ETV showed a high likelihood of HBsAg loss (22.2%) (OSST trial)^18^. In another study, 33.3% of patients receiving sequential therapy with ETV and PegIFNalfa-2a achieved HBsAg loss^24^. Recently, 15.91% patients attained HBsAg loss in the PegIFNα-2a group than in the ETV monotherapy group^21^. In this study^18,21^, NA-experienced patients with virological suppression and HBsAg levels < 1,500 IU/ml achieved higher HBsAg loss (32.6%, 41.67%) and HBsAg seroconversion (27.9%) after switching to PegIFN alfa-2b for 60 weeks than those who continued treatment with NAs (0%). The rate of HBsAg loss in this study was higher than that in the previous trials^18,21^. Extending PegIFN treatment duration (60 weeks) in this study may be an important factor for the observed superiority over 48 weeks PegIFN therapy in the previous trials. Indeed, the current study provides evidence to support the previous results that patients on long-term NAs can achieve significantly higher rates of HBsAg loss after switching to a finite course of PegIFN alfa. This finding is also consistent with previous reports showing that patients with a lower HBsAg level represent excellent candidates for the addition of PegIFN therapy and that a lower HBsAg level is associated with a high probability of HBsAg clearance and a good long-term prognosis^18,25,26^. As we all know, NK cells may play a role in the clearance of HBsAg during therapy strategies^27^. Recent data demonstrate that functional NK cell responses are restored *in vivo* administration of Peg IFNα and sustained restoration of NK cell responses is associated with an enhanced decline in HBsAg in a cohort of HBeAg positive CHB patients exposed to PegIFNα^28,29^, which was rarely achieved by monotherapy with NAs^30^. Therefore, it is no difficult to explain why higher rates of HBsAg loss were achieved after switching to a finite course of PegIFN alfa form NAs.

HBeAg loss and seroconversion are associated with an increased chance of sustained HBsAg loss^31^. The rate of HBeAg seroconversion is another important parameter for antiviral treatment of CHB. In a recent study, the rate of HBeAg seroconversion was 65.1% and HBeAg loss was 90.7% at the EOF. In one study on the efficacy of PegIFN in treatment-experienced Chinese patients, the HBeAg loss and HBeAg seroconversion rates were greater than 60%^32^.However, only 51.5% and 47% patients achieved HBeAg loss and seroconversion, respectively, after 48 weeks of PegIFN alfa treatment^20^. These results confirm that NA treatment-experienced patients with HBV DNA fully suppressed and HBsAg < 2,000 IU/ml have a greater chance of HBeAg seroconversion and HBeAg loss. However, in an ETV sequential combination therapy with PegIFN study^33^, patients with a baseline HBeAg < 200 S/CO and HBsAg < 1,000 IU/ml and an HBsAg decline at week 12 > 0.5 log10 IU/ml achieved the highest rate of HBeAg seroconversion (92.31%) and HBsAg loss (83.3%). In terms of these numbers, this study showed no extra superiority of switching from PegIFN alfa monotherapy compared to simultaneous combination therapy. Recently, more evidence has shown that sequential combination therapy with late “add-on” PegIFN alfa to ongoing NA treatment might be more beneficial^34^. Sample size and the criteria of patient entry are important influential factors that we must not neglect when comparing studies.

Recently, HBsAg levels and HBsAg declines have been considered to be an important factor associated with the response to PegIFN in CHB patients^21,35,36^. In this study, the baseline HBsAg and on-treatment HBsAg levels showed no difference between responders and non-responders. This finding is not consistent with previous reports showing that HBsAg levels are a predictor for HBeAg-positive CHB patients under long-term NAs treatment before switching to PegIFN^18^. An HBsAg decline was obvious in patients in the PegIFN switch group, and the degree of HBsAg decline was reduced after the end of PegIFN treatment. In particular, the decline in HBsAg among patients with no HBsAg loss was significantly slower than that in patients with HBsAg loss during the entire observation period. Our investigation of factors associated with treatment responses showed that only the decreases in HBsAg levels at all observation points were significantly associated with HBsAg loss in patients who received PegIFN switch treatment. This result is consistent with previous reports of HBsAg decline in CHB studies^15,21,37^. However, HBsAg decline failed to predict the responses to PegIFN treatment in a real-world hospital-based study^2^. Sample size and the criteria of patient entry are important influential factors which did not be neglected when comparing studies.

In this study, ALT elevations were observed early after switching to PegIFN alfa-2b in patients with sustained HBV DNA suppression. This finding is consistent with previous reports^18^. HBV is not directly cytopathic and the cccDNA minichromosome utilizes the cellular transcriptional machinery to generate all transcripts necessary for protein production and viral replication. Previous paper demonstrated the ability of regular IFN alfa to induce transient cccDNA suppression by acting at the epigenetic level^38^. Therefore, ALT elevations were occurred when PegIFN alfa-2b remove cccDNA. It was reported that an expanded population of activated, functional NK cells induced by a course of Peg-IFN α can be maintained for at least 9 months^29^. ALT level was still higher after termination of PegIFN alfa-2b treatment in this study.

Although PegIFN α used as a ‘switch to’ strategy achieved encouraging response in CHB patients, the strategy of switching to PegIFN α is not in favor of the recommendation by EASL 2017 Clinical Practice Guidelines on the management of hepatitis B virus infection^39^. The reasons were pointed out by EASL 2017 Clinical Practice Guidelines, as follow: The therapy of switching to PegIFN a from NAs increase cost and side effects, which should be carefully assessed in each individual patient weighing up all potential advantages and disadvantages. The evidence for superiority is lacking, and there are still many unresolved issues with respect to patient selection, timing, as well as the duration of switching strategy, which may be addressed in future studies.

Regarding safety, PegIFN switching was generally well tolerated without unexpected adverse effects. The safety profiles of PegIFN alfa-2a monotherapy were similar to those reported previously in other PegIFN and NAs sequential therapy studies. No unexpected adverse effects were reported.

There were some limitations to this study. First, the number of patients evaluated for treatment efficacy was relatively small. Second, HBV genotype data were not collected due to the low HBV DNA levels at baseline; thus, the impact of genotype on responses could not be ascertained. Previous studies have suggested that HBsAg kinetics and rates of HBeAg seroconversion vary by HBV genotype^40^. Third, the NA drugs used were varied. The aim of this study was to evaluate whether switching to PegIFN from a stable NA regimen leads to high loss of HBsAg in CHB patients with HBsAg < 2,000 IU/ml and HBV DNA < 20 IU/ml. The effect of switching to PegIFN from stable NAs was the focal point. In the future, large-scale and prospective studies are required to identify the switching strategies for patients receiving different antiviral therapies.

In conclusion, this study suggested that patients who have low HBsAg levels, despite sustained HBV DNA suppression on long-term therapy with a potent NA, have a higher rate of HBsAg loss and seroconversion and are more likely to experience HBeAg clearance by switching to PegIFN alfa-2b than from continuing NA monotherapy. In particular, the HBsAg decline should be taken into consideration to guide therapy decisions on switching to PegIFN alfa-2b. Thus, our results indicate that functional cures for CHB patients with HBsAg < 2,000 IU/ml and HBV DNA suppression can be obtained by sequential switching to PegIFN alfa-2b.

## Patients and Methods

This investigator-initiated, prospective, open-label study was conducted in the Department of Infectious Diseases of Guangdong General Hospital and the Third Affiliated Hospital of Sun Yat-Sen University between October 2013 and June 2016. Since the treatment was based on the results of the OSST trial (ClinicalTrials.gov: NCT00940485)^18^ and not approved in most of clinical practice guidelines at the time of enrolment, each participating centre obtained approval for the experimental therapy and the study protocol from a local ethics committee. This study was conducted with the Declaration of Helsinki principles for ethical research. Written informed consent was obtained from the participants, and all data were identified.

### Study population

This was an study performed in accordance with the Declaration of Helsinki. The Ethics Committee of the Guangdong General Hospital (Guangdong Academy of Medical Sciences) approved this study. Informed consent was obtained from all patients. According to the conclusion of previous study^18,21^, patients with HBsAg levels < 1500 IU/mL at baseline had higher HBeAg seroconversion and HBsAg loss rates. The aim of this study was found out whether the rate of HBsAg loss of the patients with HBsAg levels < 1500 IU/mL was raised through extended PegIFN treatment to 60 weeks. The cutoff of serum HBsAg was extended to 2,000 IU/ml to expand the sample size. Though ETV and TDF have been recommended as the first-line options for treatment of naive CHB patients, they are not used widespread in the countries with limited health resources due to the high daily cost or less available, and therefore LMV, LdT and ADV are still widely used especially in the economically less developed regions due to their low cost and easy availability. In a word, the strategy of switching to PegIFN after NAs therapy may be closer to the actual clinical practice for the CHB patients in the countries with limited health resources, which can provide clinical real response data Therefore, the inclusion criteria consisted of patients with documented HBV infection who were 18–65 years of age, had a history of NA antiviral treatment, had HBsAg < 2,000 IU/ml and serum HBV DNA levels < 20 IU/ml, HBeAg positive or negative and did not have cirrhosis (confirmed by liver biopsy or ultrasound). The exclusion criteria consisted of patients who had not been pre-treated with antiviral NAs or with immunomodulatory agents; evidence of co-infection with hepatitis A, C or D; patients with a history or evidence of chronic liver disease associated with another medical condition or decompensated liver disease (Child-Pugh score > 5); pregnancy or lactation; and any other contraindication for PegIFN therapy described in detail in a previous study^17^.

### Study design

**Figure 5:**
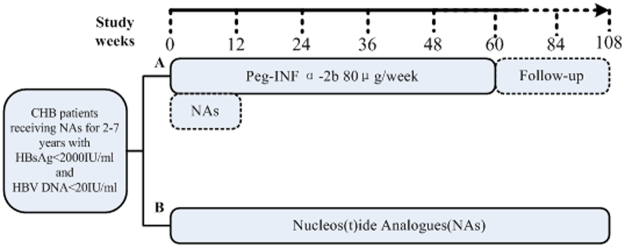
Study design. Patients in the PegIFNalfa-2b group received both PegIFNalfa-2b and NAs during the first 12 weeks. HBsAg, hepatitis B surface antigen; HBV, hepatitis B virus; CHB, chronic hepatitis B; NAs, nucleos(t)ide analogues.

Eligible patients were randomized into 2 treatment groups. Randomization was centralized, and a patient identification number assigned by the randomization system was used to allocate the patients to treatment groups. Patients in group A received PegIFN alfa-2b 1 μg/kg once a week (PegIntron, Schering-Plough, Kenilworth, NJ, USA) by subcutaneous injection for 60 weeks and continued NAs for the first 12 weeks to reduce the risk of virus rebound and fluctuations in alanineamino transferase (ALT) levels during the switching period. Patients in group B continued to receive NAs daily (Fig. 5). All patients were followed for 108 weeks. Routine blood tests were performed each month. HBV DNA, HBsAg, HBeAg, and hepatitis B antibody levels were determined every 4–8weeks. The efficacy parameters included the viral and biochemical responses. Responses were measured at 24 weeks, 48 weeks, and 60 weeks and at the 24 weeks and 48 weeks of follow-up. Viral responses included the HBV DNA loss rate, HBeAg seroconversion rate, and HBsAg loss rate. Response definitions were previously described in detail^41^.

### Definition of treatment responses

The primary endpoint was the rate of HBsAg loss at the end of treatment (EOT) (week 60). The secondary endpoint was the rate of HBsAg seroconversion (HBsAg loss accompanied by emergence of anti-HBs) at EOT (week 60).

### Laboratory assessments

HBsAg levels were quantified using the Abbott Architect HBsAg assay (Abbott Ireland Diagnostics Division, Sligo, Ireland; dynamic range 0.05–250.0 IU/ml).When HBsAg > 250.0 IU/ml, the samples were retested after the dilution of 1:500.HBeAg, anti-HBe antibodies, and anti-HBs antibodies in serum were measured with Roche chemiluminescent assays (Roche Elecsys 2010 analyzer; Roche Diagnostics, Indianapolis, IN, USA). Serum HBV DNA levels were measured centrally using the COBAS TaqMan HBV Test (Roche Molecular Diagnostics, Pleasanton, California, USA; limit of detection 20 IU/ml; testing was performed at baseline and at weeks 24, 48, 60, 84 and 108 at a central laboratory of the Third Affiliated Hospital of Sun Yat-Sen University). All patients were assessed for thyroid function, autoimmune antibodies, blood sugar, and routine blood tests before and during treatment.

### Sample Size Calculation

44 subjects in one previous reports^21^ and about 30 subjects in our previous study^42^ in each group can reveal the different effects of peginterferon alfa on CHB patients. Therefore, we decided to include 50 patients in each group which allowed for an expected 15% dropout rate in this study.

### Statistical analysis

Continuous variables were expressed as the mean ± standard deviation (SD) or median (inter- quartile) depending on the result of normality testing and were analyzed using a *t* test or Wilcoxon test, as appropriate. Categorical variables were expressed as the number of patients (percentage) and tested with the *χ2* test or Fisher’s exact test, as appropriate. A logistic regression model was conducted to determine the predictive factors for achieving a response to treatment. Receiver-operating characteristic (ROC) analysis was used to calculate the area under the ROC curves (AUC) for predictive factors. All analyses were performed as two-sided tests with a 0.05 level of significance. HBsAg levels (IU/ml) were logarithmically transformed for analysis. SPSS software (version 22.0; SPSS, Inc., Chicago, IL) was used to perform the statistical analyses.
